# Transactions of the Chicago Society of Physicians and Surgeons

**Published:** 1874-05-01

**Authors:** Plym. S. Hayes


### TRANSACTIONS OF THE CHICAGO SOCIETY OF PHYSICIANS AND SURGEONS.

### Meeting of April 13th, 1874.

*Reported by Plym. S. Hayes, M.D.*

THE Society met as usual in the parlor of the Grand Pacific Hotel, the President in the Chair.

Drs. W. H. Warn, Ralph E. Starkweather, and E. P. B. Wilder, were unanimously elected to membership.

The names of Drs. Wm. A. Harvey, G. H. Chapman, and W. M. Rofe, were proposed for membership.

Drs. Jackson, Etheridge, Merriman, and Fisher, read reports on cases of uterine fibroid tumors treated by hypodermic injections of ergotine. Dr. Jackson read reports of five cases, two of which were from hospital, and three from private practice. The tumors were intramural in the first four cases. The tumor had disappeared in one case, and in two others they are greatly diminished in size. In the first case the tumor remains about the same; but the profuse hæmorrhages, from which the patient suffered, have been diminished both in frequency and amount. The tumor was subperitoneal in the fifth case. As there was no diminution in the size of the tumor after the fifty-second injection, the treatment was discontinued.

The region of the deltoid was advised as the site for the injections, rather than the walls of the abdomen over the tumor; because the effect seemed to be as well-marked, and the operation less painful, when done in the arm. The treatment was commenced, in one case, by injections, and continued by administration of the same remedy by the mouth; the rate of diminution of the tumor being the same after the change in the mode of administration.

The following solution was preferred to that employed by Dr. Hildebrandt, as the latter produced a precipitate: Extract of ergot, 50 grains; water, 250 minims; filter, and add enough water to make 300 minims.

Dr. Etheridge reported the following case: M. B., age sixteen, had retroflexion of the uterus, and a small subperitoneal tumor of the anterior wall. The diagnosis was confirmed by Drs. Gunn and Miller. For eight weeks, nitric acid and nitrate of silver had been applied to the os, with no preceptible result. Hypodermic injections of ergotine were then used for forty days, at the end of which time no tumor could be discovered. The painful menstruation, from which the patient had suffered before this latter treatment, was entirely relieved.

Dr. Merriman reported three cases: In the first, an intramural tumor was found in the anterior wall. The treatment by ergot was continued every other day for three months, when the patient was discharged cured. In the second case, a subperitoneal pediculated fibroid tumor was attached to the anterior wall of the uterus. The patient's health has been improved by the use of ergot, and the growth of the tumor checked. The patient is still under treatment. In the third case, H. M., aged twenty-eight, and single, complained of pain in the right side and back; the bowels are regular; the menstruation is also regular, but the discharge is scanty. The tumor, diagnosed as intramural, is gradually disappearing under treatment.

Dr. Fisher read details of the following case: The patient, aged forty-seven, had retroversion of the uterus, and an intramural fibroid, which extended half way to the umbilicus. The uterus was replaced in nearly its natural position, and at two P.M. of February 4th, the injection of ergotine was commenced, and continued at the same time every day for twenty-four consecutive days, at which time the menstrual period recurred, and the injections were omitted. After this the ergotine was again used, hypodermically, for five days, and afterward given by the mouth. An examination was made in March, when the tumor was found to have disappeared, and the uterus to be in a normal condition.

In all of the above cases the use of the ergotine was discontinued during menstruation.

In reply to the question, What is the physiological action of ergotine, that causes these growths to disappear? Dr. Jackson said that the remedy acted upon the fibres of the uterus, producing contraction, which in turn causes the absorption of the neoplasm. He could not explain why the subperitoneal tumors were absorbed.

Dr. Etheridge thought there might be an action similar to that producing a cure in aneurisms, when injections of ergotine were used.

Dr. Hay thought that the drug acted directly on the arterioles and capillaries, contracting them, and preventing nutrition. The contraction of the uterine fibres was secondary to, and dependent upon, the primary or direct action.

Dr. Peck related a case in point: The patient, a lady subject to angina pectoris, was suffering from chronic metritis, accompanied by a saneo-purulent discharge, for which ergotine injections were used with benefit; but they had to be discontinued, as they produced recurrent attacks of the angina.

Dr. Jackson wished to know if there was any good reason for discontinuing the use of ergot during menstruation. He had in one case, not knowing that the patient was menstruating, exhibited the drug, during the early part of the period, with no ill effects.

Dr. Emmons reported the following case: The patient, aged forty-five, had had three children; the youngest was born eleven years previously. An examination showed that there was a fibroid tumor of the anterior wall and neck of the uterus, extending to the umbilicus. The depth of the uterine canal was six and one-half inches. The menstrual flow was very profuse, and the patient was in an exsanguinated condition. For three months prior to the first visit the patient was confined to her bed. December 31st he commenced the hypodermic use of ergotine, and has continued it to the present time without intermission. He administered the remedy during menstruation to prevent the excessive loss of blood. The tumor has not diminished, but the hæmorrhages are much less severe, and the patient is so much better that she is enabled to sit up half of the time.

Dr. Hay suggested that, as a rule, ergot should be used in these cases during menstruation, when that function became pathological, and discontinued when it was physiological.

Dr. Hollister spoke of the influence of ergot on the vaso-motor nerves, and mentioned its effect in cerebro-spinal meningitis, in proof of the statement. The following symptoms were noticed: after one drachm of ergot had been given, in meningitis, every two hours, for twenty-four hours, the cutaneous exanthem entirely disappeared; the pupils, from being greatly dilated, became normal in size; the pulse, which before its administration was 120 in a minute, irregular and thready, was reduced to 95, and without irregularity; and, finally, the intellection was clearer.

Dr. Powell said that in a case of aneurism of both popliteal arteries, that of one side was cured by digital compression, and that of the other was treated one month by injections of ergotine without result. He also stated that the use of ergotine in fibroids had not met with encouraging success in the Woman's Hospital in New York City.

Dr. Hollister mentioned that the excessive and continued use of ergot produced gangrene.

Dr. Hay thought the action of ergot in progressive locomotor-ataxia and fibroids was identical. The pathological condition found in both was due to the abnormal development of fibrous tissue, in the one case in the spinal cord, and in the other in the uterus.

Dr. F. H. Davis presented pathological specimens of a kidney, with ureter attached, which latter was of the size of a small intestine; and a section of a heart, to which the pericardium was adherent, a layer of calcareous material uniting the two. He read the report of the case from advance sheets of THE EXAMINER.

Dr. Emmons exhibited a uterus taken from the body of a woman supposed to have died from an induced abortion, which supposition, however, was proven to be incorrect, on *post-mortem* examination. The hour was so late that the case was not discussed.

Dr. Wilder requested permission to make a nomination for membership, and, on receiving it, nominated Mrs. A. P. Kent, M.D. After a short discussion, Dr. Powell made the following motion:

*Resolved*, That it is the sense of the Society, that female physicians are not eligible for membership.

A motion of Dr. Merriman, to lay the resolution on the table, was lost, and the orginal motion carried.

The Society then adjourned.

